# A Survey on Nanotechnology-Based Bioremediation of Wastewater

**DOI:** 10.1155/2022/5063177

**Published:** 2022-03-02

**Authors:** Lakshmi Thangavelu, Geetha Royapuram Veeraragavan

**Affiliations:** ^1^Department of Pharmacology, Mandy Dental College and Hospital, University of Dhaka, Dhaka, Bangladesh; ^2^Centre for Transdisciplinary Research, Department of Pharmacology, Saveetha Dental College and Hospitals, Saveetha Institute for Medical and Technical Sciences, Saveetha University, Chennai 600077, Tamil Nadu, India; ^3^Department of Microbiology, Saveetha Dental College and Hospitals, Saveetha Institute for Medical and Technical Sciences, Saveetha University, Chennai 600077, Tamil Nadu, India

## Abstract

Rainwater discharge and human impacts produce wastewater, which is a contaminated type of water. Sediments also discharge phosphate into the water column when there is a lack of dissolved oxygen in the water. Through the manufacturing of environmentally benign nanoparticles, nanotechnology may reduce the amount of money spent by enterprises to remediate such contaminants. Because of their improved physiological, biochemical, and biomechanical qualities, nanoparticles are getting prominence. The importance of the global wastewater dilemma is discussed in this survey. The use of nanomaterials in heavy metal remediation (HMR) and wastewater treatment is covered in this survey. This paper also discusses the benefits of nanotechnology over traditional approaches in certain fields. This survey aims to gather together many recent studies on nanoparticle production and their benefits as adsorbents in the remediation of wastewater which have been done so far. The promising role of nanotechnology in wastewater remediation is surveyed in this research, which also discusses recent developments in nanotechnology-mediated remediation methods. This survey examines the vital potential of nanotechnology in wastewater treatment, as well as recent breakthroughs in nanotechnology-mediated treatment systems.

## 1. Introduction

The most significant water sources for household and industrial purposes are groundwater and surface water. 70% of the accessible groundwater is used for agriculture activities. It is also the most plentiful source of drinking water. Water is needed for livestock and cultivation in agriculture, but it also has a lot of functions in manufacture, including chilling, cleaning, refining, transportation, and dissolving. Water is also critical for an industry's hygienic demands, as effluent including xenobiotics is dumped into nearby rivers or sewers. Approximately 300–400 megatonnes of various dangerous pollutants, including dyes, heavy metals, sludge, and other garbage, are released directly into water systems each year. Every year, the globe consumes around 7 lakh tonnes of different extremely carcinogenic pigments and dyes [1]. As a result, dumping these poisonous chemicals without adequate treatment may cause severe health problems. Heavy metals (HM) are also very toxic and may dissolve in water, poisoning it. Information on groundwater contamination with HMs in the rural areas of India in recent years revealed that average concentrations of manganese (Mn), arsenic (As), lead (Pb), chromium (Cr), zinc (Zn), and nickel (Ni) in drinking water are higher than the threshold limit suggested by WHO [2]. Programs to determine the water quality are becoming necessary to ensure the public health of vulnerable water sources [3].

Diverse human activity in companies, markets, agricultural areas, and everyday domestic behaviors generates massive amounts of wastewater. Both human health and the ecosystem are endangered by this pollution. Worldwide, the population is increasing at a steady rate, and towns are becoming overcrowded and reaching load capacity. To fulfill the increased need for social beings, enterprises are expanding at a quicker rate, generating far more effluent than before, further complicating the issue. The dairy business, as a major agro-based sector, heavily contributed to industry-related water contamination. Dairy effluent comprises a significant organic pollutant, as well as odorous chemicals and micronutrients. Sedimentation, screening, oxygenation, filtering, and other physicochemical procedures have all been investigated extensively for treating wastewater. However, biological approaches are a superior wastewater treatment option owing to constraints including partial treatment, increased price, formation of secondary pollutants, significant solid deposition, and usage of different chemicals [4].

Wastewater contains components such as phosphorus, nitrogen, and carbon that may encourage the development of unwanted organisms in the marine ecosystem. Dissolved inorganic elements such as salt, calcium, suspended particles, biodegradable compounds, microorganisms, and heavy metals are also present. This problem can be resolved through wastewater treatment. HM reduction is one of the most difficult issues to solve. The conventional techniques applied for HMR have several drawbacks. Physical removal of organic pollutants is a common part of traditional cleanup procedures. Physical cleanup techniques are inefficient and often cause environmental disruption. We need an effective approach for removing heavy metals to address the existing predicament [5]. Nanotechnology is the alternate technique for removing heavy metals from contaminated bodies. It is a burgeoning industry that is being coupled with the most common conventional ways for eliminating HMs from wastewater [6]. Nanomaterials are being defined by the International Organization for Standardization Technical Committee 229 (Nanotechnologies), which is producing an internationally agreed nomenclature and terminology. Nanoparticles are described as materials having one, two, or three exterior dimensions in the size range of roughly 1–100 nm, according to ISO/TS 27687:2008. Such substances are capable of reacting with chemicals and impurities. They can penetrate extensively into pollutants, enhancing their responsiveness and, as a result, their effectiveness in removing impurities [7].

Bioremediation is the act of optimizing naturally existing remedial activities that require living creatures to degrade, alter, or remove harmful organic contaminants. This biological method relies on microorganisms' catabolic processes and their potential to contribute to the breakdown of organic pollutants when they are used as a supply of nutrition and energy. The use of bioremediation methods to dispose of untreated wastewater during crop irrigation is an alternative; however, the effectiveness of bioremediation methods is dependent on a variety of aspects to consider when selecting a treatment, which offers the quality of the water, satisfying the requirements of plants. Firstly, the pollutants' characteristics are assessed, as these determine their biodegradation capability, as well as any adverse effects that could impact the areas from which pollutants must be eliminated [8].

## 2. Toxic Impact of Heavy Metals

Heavy metals are defined as substances having a concentration greater than 6.0 g/cm3. HMs have a significant biological impact on the operations of animals and plants but only at levels underneath the WHO-approved standard intake levels. The incorrect disposal of heavy metals is a significant pollutant source in the industrialized and developing nations [9]. Industrial and commercial operations are the primary sources of heavy metals in wastewater [10]. Those that enter the environment through natural procedures like forest fires and volcanic outbursts are often less destructive compared to those that enter through manmade sources like mines, smelters, and foundries [11]. They are one of the most common contaminants in wastewater, and they are harmful to the plants, environment, people, and aquatic life. Anthropogenic activities like mining, untreated industrial wastewater discharge, and the use of pesticides and fertilizers containing heavy metals in agricultural operations all contribute to heavy metal pollution [12]. Higher quantities of heavy metals may harm cell membranes, limit seed viability, lower pollen grains, and negatively impact the fora and fauna. In nature, they are very poisonous and nonbiodegradable [13]. They have a great affinity for the same binding sites that important metal ions employ for diverse cellular structures. This leads to destabilization, which leads to replication errors, cancer, and mutagenesis [14]. Heavy metals affect a variety of physiological and biochemical functions, and they not only injure cells by increasing the number of free radicals but also denature microorganisms. They may also impair microorganisms' bioremediation ability [15]. The common mechanism of toxicity caused by heavy metals is explained as follows. These compounds react with biomolecules when they are consumed by humans. During interactions, oxidative stress may develop due to the scarcity of biomolecule antioxidants. This increases the production of reactive oxygen species (ROS) such as H_2_O_2_, O_2_, and hydroperoxides [16]. The increase of ROS produces lipid peroxidation, which may harm the plasma membrane. ROS may damage enzymes, nucleic acids, and lipids, impairing normal cell activity and perhaps leading to cell death [17]. Heavy metals interact with substrates, preventing key enzymatic processes and altering enzyme structure [18]. Heavy metals are also known to produce ion imbalance owing to adhesion to the surface of cells and penetration through carriers or channels [19].

## 3. Existence of Heavy Metals in Wastewater

**Figure 1:**
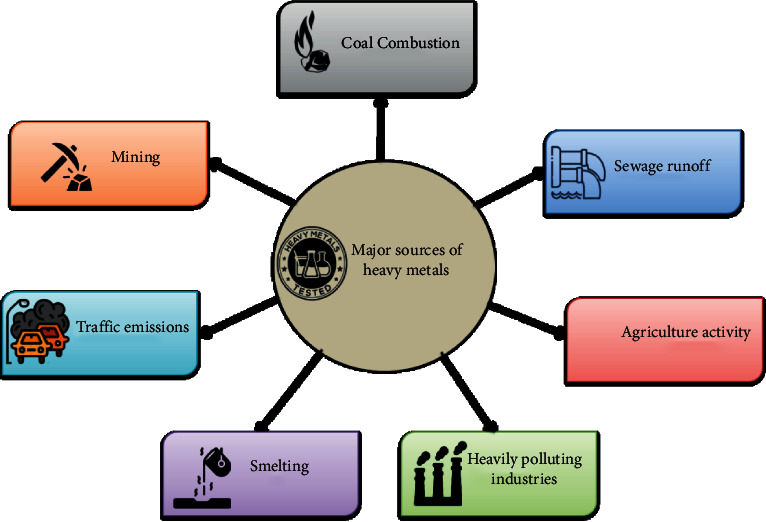
Sources of heavy metals.

Wastewater is largely polluted by HMs, which is generated due to mining activities, combustion of coal, smoke emitted during traffic, agriculture activities, sewage runoff, and heavily polluting industries such as foundries, smelting, petrochemical, plastics, painting, textile, fabric, printing, ceramics, batteries, paper-pulp, fiber, pharmaceutical, and chemical industries [20].  Figure 1 depicts the major sources of heavy metals.

Likewise, [21] suggested a list of large industries such as metalliferous mining industries that release acid mine tailing and drainage containing HMs, manure sewage sludge having HMs, fertilizers sectors that emit HMs in surface and groundwater, alloys and steels sector that produce, discard, reprocess metals, tailings, and slag heaps, and emit HMs, paints and pigments that emit aqueous waste from the production, old paint deterioration and soil pollution having HMs, electroplating sectors that emit liquid affluents from plating procedure having HMs, and waste disposal discharge landfill leachate, polluting ground and surface waters including HMs.

## 4. Destructive Properties of Heavy Metals

In the food web, HM moving from water to plants and people may pose a danger to ecosystems. Drinking water contaminated with HM may cause serious toxicity and illness in humans [22]. The higher pollutant range for HMs in drinking water is Hg (0.003 ppm), Pb (0.016 ppm), Cr (0.2 ppm), Cu (1.3 ppm), Cd (0.006 ppm), and Zn and Ni (0.05 ppm), according to the US EPA (2009). When concentrations of these heavy metals exceed safe levels, they may harm aquatic life, humans, and the soil's fertility [23]. The common adverse effects of HMs on human health are reported in Table 1. Several illnesses and syndromes may be caused by heavy metal exposure like Parkinson's and Alzheimer's disease as well as liver impairment [34].

## 5. Conventional Methods for Wastewater Treatment

The use of time-tested techniques for treating sewage, the economy, and the environment all play a role in wastewater treatment. Before creating any technique, these considerations are taken into account. Heavy metal elimination needs both immersion and isolation due to the difficulty of removing these pollutants using biological, physical, or chemical methods [35]. Ion exchange chemical oxidation, reduction, and precipitation, photocatalysis, membrane filtration, adsorption, reverse osmosis, and electrodialysis are some of the typical procedures now in use. To treat wastewater contaminated with heavy metals, researchers used a variety of approaches, including conventional, microbial, plant-based, and nanomaterial-based techniques.  Table 2 provides a detailed description of different techniques available for the elimination of HMs from wastewater. This table presents the comprehensive differences and highlights the ways to improve current techniques in water treatment. These approaches have several advantages, including their ability to be controlled and their capacity to withstand high levels of heavy metals. Nitrogenous and carbon components are oxidized using suspended bacteria in the activated sludge process to generate an effect that is within regulatory norms and causes minimum environmental impact [40]. Convenience and electrostatic attraction are essential factors in adsorption in polymer adsorbents, regardless of whether chemisorption or physisorption takes place [41]. It is difficult to dispose of the waste products produced by conventional processes, and they consume a lot of energy. Because the materials used in these processes are derived from nonrenewable resources, they may be hazardous to the environment. Due to the high expense of physiochemical procedures, they cannot be used in impoverished and underdeveloped nations. As a result, these approaches deplete soil fertility, rendering them unsuitable for agricultural use [42]. High energy use, inadequate pollution removal, and harmful byproduct formation are some of the problems with traditional technologies. The employment of microbial methods along with physical procedures as a bioremediation strategy may lead to better removal of HMs from wastewater [36].

## 6. Nanoparticles Based Advanced New Approaches for Heavy Metal Elimination

Innovative methods and materials for the identification and removal of particular HMs are also being developed by scientific groups [43]. The elimination of pollutant and hazardous chemicals from wastewater is another use for nanocomposite or nanoparticles. As an example, a minimized graphene-iron oxide nanocomposite adsorbent for the elimination of phenazopyridine was developed [44]. Heavy metals may also be removed with this method. Thus, nanomaterial technology has been used to enhance the elimination of a variety of harmful compounds [45]. As an additional benefit, the ligand-dependent functional material is ideal for eliminating heavy metals and other pollutants from wastewater. The ligand-based functional material consists of a variety of composite materials, each of which has a specific organic functional group affixed to the carrier. When compared to unmodified ion exchange materials, it has great adsorption capacity and a high degree of selectivity for metal ions [46]. To remove HMs from polluted water, organic ligand-based composite materials may be used. To eliminate HMs using a ligand-based method, the pH, identification, and reaction time are all critical [46]. Reference [47] employed an embeddable composite adsorbent to remove Ni(II) from wastewater polluted with petroleum products. Mesoporous silica was used to attach the dimethylglyoxime-based composite adsorbent. The adsorption capability of 198.42 mg/g1 of dimethylglyoxime ligand composite was determined using Langmuir's adsorption isotherm equation. Another study assessed the removal of Ni from the organic ligand-based composite materials [46]. Their mesoporous silica monoliths were prepared using Tetramethyl orthosilicate, Pluronic F108, HCL, and water (4 : 1; 2; 1). They dried the material at 45°C for 24 hrs. Direct ligand immobilization of 2-nitroso-1-naphthol was used to create the composite material. According to the results, the identification range for Ni(II) was 0.41%, which means that raising the pH may enhance the ion's removal potential. However, the highest adsorption capacity, 199.19 mg g^−1^, was attained at pH 7 for the identification and elimination of Ni(II).

A direct anchoring approach was used to create a facile composite material with organic ligands and larger pores [46]. To eliminate Pb(II) from the aqueous solution, this ligand system was utilized.

At a pH range of 5–50, the highest adsorption capability was detected to be 176.66 mg g^−1^. For the identification and elimination of Co(II) from aqueous samples, [46] developed a composite mesoporous silica adsorbent with a functional ligand (3-((5-ethoxy benzene thiol)aminomethyl)-salicylic acid). Because of its amino-salicylic acid-base and ability to detect minimum levels of Co(II), the ligand may detect concentrations as low as 0.39 g L^−1^ using the 3-(((5-ethoxy benzene thiol)imino)methyl). 185.23 mg g1 was the ligand's highest adsorption capacity. In addition, [46] developed an organic ligand contained in inorganic-organic mesoporous composite elements for the identification and elimination of V.(III) [46].

Additionally, the nonfunctional combined material can identify contaminants in wastewater. For example, [46] produced porous conjugate material functionalized with a 4-nitro-1-naphthylamine ligand to investigate the elimination of NO_2_ from water samples. The greatest adsorption capability of this combined element was 124•36 g L^−1^ of NO_2_. These conjugates may also be utilized to eliminate heavy metals from water sources. When it comes to cesium removal from water sources, for instance, they developed an improved combination of elements (ligand attached). Acetyl dibenzo-20-crown-6-ethers were produced and introduced into mesoporous inorganic silica, where they studied the effects of pH, starting cesium concentration, and contact time on the macrocyclic ligand. For the highest adsorption capacity (65.06 mg L^−1^), pH 7 was found to be the best, according to the results. Because of its great selectivity for certain heavy metals, this ligand-dependent nanomaterial sensor technique is a potential tool for wastewater treatment [46]. A synthetic zeolite-based adsorbent was also created by [48] to remove cesium from simulated wastewater. Using hydrothermal alteration, zeolite adsorbent was created from the molten slag of municipal wastewater sludge. The cesium removal efficiency was found to be 97.36 percent at thermodynamic constants of 308k. Hydrothermal treatment may therefore be able to increase the zeolite content in bio slug, which may in turn help remove radioactive and heavy metals from wastewater.

Heavy metals in contaminated water have been detected using chelating agents. A variety of chelating compounds have been utilized, including N, N'-ethane-1,2-diyl)bis(2,5 dimethoxybenzene sulfonamide), and E-4-methyl-N'-(1-(pyridin-2-yl)ethylidene) benzene sulfonohydrazide. Both N'-[1-pyridin-2-yl) ethylidene] benzene sulfonohydrazide and N'-[1-(Pyridin-2-yl)ethoxy] were shown to be efficient in the detection of Hg(II) [49]. Researchers found that N,N'-bis (4-methoxybenzene sulphonamide) and (ethan-1,2-diyl)bis (3,4-dimethoxybenzene sulfonamide) can detect Ni(II) ions, and chelating agents may aid in the identification of certain HMs from water [49]. The carbon electrodes that are glassy in nature, silver oxide, and zinc oxide were employed in the construction of an electrochemical sensor that can detect heavy metals; these metal oxides were combined to create the sensor. The wet chemicals (coprecipitation) approach was used to manufacture the extremely sensitive sensor, which can detect heavy metals and dangerous compounds including xanthine, 2-nitrophenol, and hydrazine [34]. For the identification of 4-4-hexyl resorcinol, [49] created a nanorod-based sensor that was made utilizing cobalt oxide and the conjunction of erbium oxides below the reduction of alkaline media. Additionally, [49] employed a wet chemical approach to manufacture silver oxide nanosheets including lanthanum oxide nanosheets. To remove 3-methoxy aniline, the authors used a Nafion glass carbon electrode modified with silver oxide-lanthanum oxide nanosheets and a 5% ethanolic binder to create the glassy carbon electrode. This resulted in a highly selective electrochemical sensor. As a result, sensors for heavy metal detection might be designed using these methods. On the other hand, [45] created a slurry of (E)-N′(2-nitro dolamide)-Benzenediaminesulfohydrazide, which was then applied to the carbon electrode with a binding agent, Nafion, to create an extremely sensitive HM sensor [45].

## 7. Gold-Based Nanomaterial in Heavy Metal Elimination

Heavy metals are being identified and removed employing nanomaterials in a variety of applications. Gold is a superb option for extracting toxic metals as a nanomaterial. Gold nanomaterials have been shown to be effective in eliminating toxic metals and have good selectivity for a range of different species [50]. The impact of AuNPs of various shapes and sizes on the elimination of Hg^2+^ was analyzed [51]. The lack of surface treatment of AuNPs (gold nanomaterials) has been shown to affect reusability, as they are likely to clump into groups [52]. This problem can be overcome by isolating them on Al_2_O_3_ surfaces. The adsorption capabilities of AuNPs-poly(dimethylsiloxane) nanocomposite foam can be accelerated from 0.28 to 4.066 by utilizing NaBH_4_ as a reducing agent for Hg^2+^ [53]. AuNPs-poly(dimethylsiloxane) nanocomposite foam has a 6-fold maximum extraction capability against organic materials in water compared to poly(dimethylsiloxane) foam without AuNPs. With a dispersion constant of 0.4 nM, gold nanomaterial adsorbents have a typical binding affinity for Hg^2+^ ions, although Al_2_O_3_ adsorbents have a somewhat lower dissociation constant of 53.9 nM. The hybrid adsorbent that contains gold nanoparticles and Al_2_O_3_ has a great affinity for mercuric compounds and some other metal ions [54]. This may be due to the synergic impact. The AuNP–Al_2_O_3_ adsorbent has a mercury elimination rate of about 96 percent, and the approach is expensive, efficient, and reliable [55].

## 8. Iron-Based Nanomaterial in Heavy Metal Elimination

High-arsenic absorption capacity was observed in nanocomposites composed of iron oxide enclosed in macroporous silica (FexMOSF). Iron-dependent composites adsorb 47 times over other nanoadsorbents [56]. Iron may act as an adsorbent for HM ions in wastewater. Because iron compounds have large specific surface areas and strong binding energies, they are effective HM adsorbents. By adsorption mechanism, most of the iron-based nanomaterials remove HMs from wastewater. Nanoscale hydrated iron (III) oxide (HFO) materials have a maximum sorption affinity for both kinds of arsenic, and the necessary contact time is likewise quite short (4 min) [57]. Reference [58] discovered that maghemite nanomaterials are more efficient than magnetite derivatives nanomaterials at eliminating chromium from the aqueous solution. As a consequence, there is an extremely little conflict for associating with chromium compounds against commonly occurring ions in water including sodium, magnesium, nickel, chlorine, copper, calcium, and nitrates. The impact of Fe_3_O_4_ in the removal of Pb ions from polluted water was investigated [59]. Pb (II) had the maximum adsorption capability, measuring 37 mg/g. The hydrothermal application of Fe_3_O_4_ superparamagnetic nanomaterials covered with ascorbic acid indicated effective arsenic elimination from wastewater [60]. The higher adsorption capability found for As (III) was 16.57 mg/g, whereas As (IV) had 47.06 mg/g (V). In the case of Pb ions, Fe_3_O_4_ has a higher adsorption capability of 84 mg/g, as per analysis [61]. Metal oxide NPs have a less magnetic behavior as a result of the coprecipitation technique, which made them easily detachable utilizing magnetic fields. Fluoride was eliminated from the water supply utilizing an Fe-Ti bimetallic oxide wrapped magnetic Fe_3_O_4_ nanocomposite [62]. Nanomaterials were produced utilizing the coprecipitation technique, and it was found that these nanomaterials had a maximum adsorption capability of 58.23 mg/g.

## 9. Silver-Based Nanomaterial in Heavy Metal Elimination

According to many findings, nanomaterials can eliminate heavy metals. There are various studies in the literature that demonstrate that nanomaterials react with pollutants including mercury, cadmium, and chromium. The utilization of silver nanomaterials comprising mercaptosuccinic acid and assisted by enabled alumina for mercuric ion removal from pollutant waters was described in a study. Silver nanoparticles were discovered to have higher mercuric ion absorption efficiency. Reference [63] utilized silver-supported nano mesoporous silica for mercury removal from wastewater and discovered that the nanomaterial was effective in absorbing mercury ions. Another study indicated using zero-valent Ag nanomaterials for efficient cadmium elimination which can be generated utilizing *Ficus benjamina* leaf extract. Whenever the amount of nanomaterials is raised, the elimination efficiency improves [64]. The authors therein also used *Piliostigma thonningii* leaf extract to generate Ag nanomaterials and evaluate its potential role in heavy metal elimination from lab setting effluent. Reference [65] reported on the impact of nanomaterials infused cotton in the elimination of Hg, Ni, and Cr ions from polluted wastewater and discovered that mercuric ions had the largest adsorption capacity on nanomaterial surfaces. The authors therein made nanomaterials from the gums of *Azadirachta indica*, *Araucaria heterophylla*, and *Prosopis chilensis* and revealed that they could be utilized to eliminate chromium. In that other study, Ag nanomaterials were produced in a similar manner utilizing *Prosopis julifora* leaf extract and coated with chitosan. Copper ion absorption was found to be 82 percent in chitosan enclosed nanomaterials.

## 10. Titanium-Based Nanomaterial in Heavy Metal Elimination

Due to its stable and harmless quality, TiO_2_ has a wide variety of uses in the industry, extending from cosmetics to heavy metal treatment. It also has a steady recombination process and excellent crystallinity with a low bandgap, making it ideal for bioremediation. TiO_2_ may also eliminate lead particles with an adsorption capability of 158 mg/g, according to research. Ti adsorbent had the adsorption capacity for copper, lead, and arsenic in research, with pH raising the adsorption capability for lead and copper. The use of mesoporous hybrid particles having ZnO and TiO_2_ led to a greater surface region, and the total expense of the adsorption mechanism was minimized since the nanosorbent can be recycled up to three times owing to its minimized form [66]. Rather than only reducing, trapping, or isolating the pollutant, TiO_2_ may destroy or diminish it through a photocatalytic method. The use of TiO_2_ as light-responsive component to treat polluted wastewater has gained the interest of several young researchers. When bombarded with light, it may release potential free radicals that are capable of degrading a wide spectrum of organic pollutants and minimizing HM ions.

## 11. Cerium Based Nanomaterial in Heavy Metal Elimination

CeO_2_-CNTs can eliminate arsenic anions, and arsenic-loaded CeO_2_-CNTs can be quickly and efficiently produced, as per research. At a normal pH limit, CeO_2_ nanomaterials may effectively eliminate chromium ions from water. Dispersed cerium oxide nanomaterials maintained with hexamethylenetetramine were used to eliminate chromium (VI) from contaminated water and it was indicated that they might also be used to treat wastewater. As per the research, CeO_2_ nanomaterials had a greater Pb (II) elimination efficiency than Fe_3_O_4_ and TiO_2_. CeO_2_ has the disadvantage of enhanced phytotoxicity; however, TiO_2_ and Fe_3_O_4_ NPs have no such toxicity. In research, cerium oxide nanomaterials were utilized as nano adsorbents in both single-component and multicomponent aqueous solutions to effectively remove lead, cadmium, and chromium from aqueous solutions. The adsorption capability of lead was not affected by pH, although Cd and Cr were damaged. The greatest adsorption capabilities were 94.4 mg for cadmium at pH 7.1, 129.1 mg for the lead at pH 5.1, and 35.4 mg for chromium at pH 5 [52].

## 12. Copper-Based Nanomaterial in Heavy Metal Elimination

**Figure 2:**
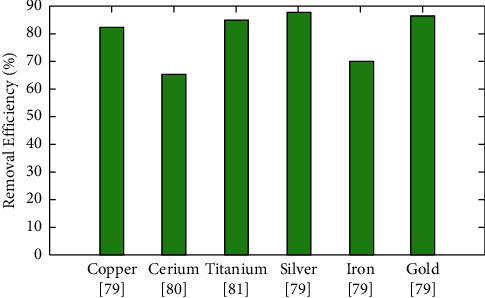
Comparative analysis of removal efficiency (%) for various nanomaterials.

The best adsorbent amount for eliminating cadmium (II) and nickel (II) ions was 0.2 g, as per the experts, and, after this elimination, the percentage increased somewhat. As per research, copper works as an excellent adsorbent of Pb (II) in polluted water, with improved elimination efficiency because of the porosity and great surface region. Due to their larger surface area and porosity, which considerably enhance the number of available active sides and the functional groups, copper nanoflowers have outstanding adsorption of Pb (II) in an aqueous solution [67]. As per research, carbon mixed with silver-copper combined oxides has higher removal efficiency for Pb and Fe. It was observed that as the number of copper oxide nanomaterials increases, the effectiveness of HM elimination also increases. This could be related to a rise in the surface region for adsorption [68]. The comparison of removal efficiency for different types of nanomaterials is provided in Figure 2.

## 13. Applications of Nanomaterials in Wastewater Treatment

Nanomaterials are extremely small molecules that show quantum impacts by clustering their electrons collectively. Due to their size, they have a variety of unique and apparent features. These have applications in different fields, such as photonics, catalysis, electronics, and biology [69]. Nanomaterials can have a significant variety of features in comparison to the bulk materials, enabling us to build novel materials with diverse uses in the industry. By employing a single-stage treatment method that can remove a range of contaminants present in pollutant water, nanoparticles can make wastewater treatment more power-efficient [70]. In treating wastewater, nanomaterials are utilized as adsorbents. According to various studies, nanomaterials' structural qualities including such good specificity and adsorption capability make them useful at eliminating heavy metal ions from wastewater at minimum concentrations [71]. These are excellent for the adsorption of toxic substances or other pollutants because of their elevated surface region-to-volume proportion [72]. Nanoparticles have easily entered further, enhanced reactivity, and eliminated toxic metals more efficiently [73]. The recent studies are focusing on the possible use of particles such as nanocomposite, carbon nanotubes, nanofibers, nanospheres, and nanowires in conjunction with traditional wastewater techniques to help remove different organic and inorganic pollutants, particularly heavy metals [74]. The number of heavy metals available, as well as the exterior surface region accessible, impacts the diffusion capability of nanoadsorbents. Dispersion on the adsorbent's pores precedes diffusion on the adsorbent's outer surface [41]. The adsorbent size, shape, grouping condition, surface chemistry and fractal dimensions, solubility, and crystal structure are all aspects that influence the properties of nanoadsorbents [75]. Nanoparticles, like bulk materials, enable atomic-level changes, going to open up a slew of new properties not available in bulk materials [76].

## 14. Nanomembrane Technology in Wastewater Treatment

Chemical, evaporation, ion exchange, freezing, membrane ultrafiltration, sedimentation, and electrochemical processes have all been used to remove HMs from wastewater. However, none of them were completely effective owing to the high cost of removing low concentrations of heavy metals and creating sludge. These factors have led to the development of innovative wastewater treatment technologies such as nanomembrane. Nanomembranes made of nanofibers are promising options for removing unwanted HMs in wastewater treatment processes [77]. Nanomembranes may be extremely selective in the production of freshwater and are crucial in the treatment of contaminants. These membranes are porous thin-layered membranes that are impervious to HMs, salt, bacteria, and other contaminants. The membranes can be used to remove HM in a variety of ways such as membranes with surface charges for HM ion repellent, adsorptive membranes, membrane distillation, HM removal by size exclusion, and more. But adsorptive membranes and size exclusion removal are the two most common methods for removing HM from waterways using membrane-based remediation. Membranes often sieve molecules according to their size. Only particles with a diameter greater than the pore size are kept. Because the pore size of typical ultrafiltration (UF) membranes is big and would enable HM ions to pass through, the removal of HM needs the use of nanofiltration (NF) membranes in this scenario. The general premise of nanomembranes is that undesired pollutants are removed by filtering. The usage of nanomembranes makes the treatment procedure go quite quickly.

Nanofiltration (NF) is a membrane-based process. It uses pressure to segregate pollutants from water streams and to keep delayed solids out of a contaminant-enclosing water stream. Viruses, bacteria, suspended particles, dissolved organics, large multivalent ions, herbicides, pesticides, and other contaminants may all be removed. It is more efficient than microfilters and ultrafilters, yet it consumes less energy. Also, the cost of operating NF is less. In addition, the treatment plant takes less space and is simpler to install and operate than traditional treatment methods. A fraction of the feed passes through a semipermeable membrane during the membrane filtering process. The intake stream is divided into permeate and retentate at this point. The filtered component of the stream is termed as permeate, while the nonfiltered portion is retentate. Softening and elimination of natural and synthetic organic matters have been widely documented by NF. Reference [78] developed a unique nanofiltration approach that employs electrospun nanofibrous membranes (ENMs) with iron oxide nanoparticles for successful oil spill cleanup in water. Reference [79] treated dye-containing wastewater using a cross-linked polyetherimide- (PEI-) dependent NF. NF membrane was based on m-phenylenediamine. Reference [80] used Fe-modified montmorillonite (MMT) nanomembrane as an adsorbent material for cleaning wastewaters containing Hg in aqueous solutions in combination with cyanide.

## 15. Nanomaterial Based Removal of Organic Pollutants and Dyes

With the advancement of civilization, organic compound pollution in water has become a pressing issue that must be addressed. Organic chemicals found in water include dye effluent, petroleum wastewater, antibiotics, and pharmaceuticals, all of which have harmed human health and societal development. The chemical activity and adsorptive capacity of the surface metals of the nanoadsorbents were boosted by nanoparticles due to the larger surface area. The most widely used adsorbents for removing HM ions and organic dyes from aqueous solutions are carbon and its derivatives. Many studies have been interested in using carbon nanotubes- (CNTs-) based materials to degrade organic contaminants in organic wastewater treatment [81]. Nano copper oxide generated from e-waste, mesoporous silica nanomaterial, chitosan nanomembrane, sulphuric acid-treated magnetic chitosan nanoadsorbents, multiwalled CNTs, and chitosan nanoadsorbents are some of the nanomaterials that have been used to remove the dyes from wastewater lately.

## 16. Economical Factors of Remediation Procedure

Rehabilitation processes rely on socioeconomic repercussions in addition to societal expenses and operational specifics. Because nanotechnology is a new field that has just been established in the past years, assessing the financial implications of nanoremediation was not yet possible. Nanotechnology influences both the business and academic sectors if well-defined approaches and techniques are used. Analyzing the financial implications of the procedure will assist decision-makers and others in developing the methodology and products used [82]. Nanotechnology, like most other treatment processes, is an environmentally benign, resilient, and financially feasible approach for decontaminating dirty water bodies [24]. According to official analysis, using activated carbon in a marketing context is not practical. Alternatively, we may use the same substitute tool to build the procedure extra cost-efficient and to allow for a larger range of regenerative behavior.

## 17. Conclusion

The status of heavy metal bioremediation, as evaluated throughout this article, has a lot of promise in terms of metal biosorption and detoxification. This study indicates that nanotechnology has good prospects in heavy metal cleanup. Nanomaterials research and breakthroughs in this discipline aid in the discovery of new cleanup procedures. Nanomaterial architecture allows us to change the material's characteristics while improving the affinity, capability, and selectivity of contaminants. This will result in fewer hazardous compounds being discharged into the atmosphere. Nanotechnology-based remediation solutions can be safer because they do not require the use of hazardous chemicals like chlorine and ozone. Various nanomaterials could be investigated for their potential as heavy metal adsorbents. Based on the findings of the literature review, nanomaterials can be used to maximize the removal efficiency in bioremediation procedures. Special attention should be paid to the recycling, renewal, and recovery of nanomaterials to enhance the cost-effectiveness of nano-based bioremediation. These must be created in such a way that their ecological toxicity and dangers are reduced, and the like catalysts must be designed which offer minimum or no danger to the environment.

The future scope will allow the creation of low-cost, high-efficiency nanocatalysts for real-world applications. Despite the great advancements of nanoparticles in wastewater cleaning, no real-time monitoring methods for continuous evaluation of the augmented nanomaterial in the treated wastewater system were provided. As a result, one of the next efforts might be the creation of real-time models to forecast the destiny of nanoparticles throughout the treatment process, which could aid in a better understanding of the removal process and the safe disposal of nanomaterials. Although nanomaterials are effective in treating wastewater, they have had certain drawbacks like fouling issues, low resistance, and stability of membrane for a long time. So, research on new-generation versatile nanomaterials that can overcome these issues and that can be reused potentially must be carried out. Furthermore, biosafety associated with the use of nanoparticles is a major problem owing to a lack of understanding and proven methodologies for assessing the effect of these materials on human health, biodiversity loss and bioaccumulation, and NM movement in trophic chains. By addressing these difficulties, the potential for the reliable use of nanomaterials in wastewater treatment will be expanded.

## Figures and Tables

**Table 1:** Effects of heavy metals on human health.

S. no.	Reference	Materials	Definition	Effects
1	[24]	Copper	(i) Drinking water (ii) Food	Acute gastrointestinal problems, liver damage, and newborns are all possible side effects. Cr-(VI) is a carcinogen classified as category one.

2	[25]	Cobalt	Naturally occurring in a variety of forms and a component of a variety of manmade sources. Other forms of exposure include occupational, nutritional, and medicinal consumption.	Hematological and endocrine disorders, as well as failing MoM hip implants, have all been reported.

3	[26]	Europium	(i) Rare Earth metals (ii) Nuclear rods (iii) Anthropogenic origins	Metal compounds containing europium may generate fire and explosion dangers when inhaled as dust. The fatal values of europium nitrate and europium chloride are quite high.

4	[27]	Lead	(i) Water (ii) Toys (iii) Paint (iv) Folk medicines (v) Dust (vi) Cosmetics (vii) Soil metal costume jewelry and occupational origins.	Nervous system illnesses, blood problems, etc. Effects on the kidneys and brain, as well as cognitive and behavioral problems, increased oxidative stress, and interference with the central nervous system.

5	[28]	Chromium	(i) Chromium-containing road dust (ii) Wood preservation (iii) Metal treatment (iv) Oxidative pigment (v) Fossil fuel combustion (vi) Oil drilling locations	The lungs, as well as the liver, skin, immune system, and kidneys, may be harmed.

6	[29]	Arsenic	(i) The trivalent atomic state is found with other metals. (ii) Deep well water (iii) Pesticides (iv) Coke oven emission (v) Mining sites of all these examples of contaminants found in soil and water.	Skin lesions, perceived neurological deficits, impairments to the central nervous system (CNS) in children, and oxidative stress are all risks associated with aging.

7	[30]	Tin	(i) Seafood (ii) Meat (iii) Anthropogenic sources	In addition to anemia and abdominal discomfort, divalent tin salts induce gastrointestinal irritation.

8	[31]	Cadmium	(i) Soil, sewage, sludge, battery, plating, air, water	Impact of cell growth, differentiation, and apoptosis. Inhibits the activity of antioxidants and enzymes aggregates in humans, nephrotoxicity. kidney and liver

9	[32]	Nickel	The Earth's crust and core are rich in this mineral. Occurs in the environment, including the (i) air (ii) water (iii) sediment	Cancer risks in the lungs. Epigenetic impact, contact dermatitis, headaches, gastrointestinal (GI) symptoms, respiratory manifestations, lung cancer.

10	[33]	Mercury	(i) It exists in water, air, and soil. (ii) Available in three forms, namely, inorganic mercury (Hg^+^, Hg^2+^), elemental or metallic mercury (Hg0), and organic mercury like methyl or ethyl mercury	Renal dysfunction, GI ulceration, hepatotoxicity, and central nervous system damage.

**Table 2:** Comparisons between several HMR methods.

S. no.	Reference	Methods	Structure	Merits	Demerits
1.	[36, 37]	Conventional method	(i) Chemical oxidation (ii) Reverse osmosis (iii) Ion exchanger (iv) Adsorption (v) Reduction	(i) High controllability (ii) Resistance to a big number of heavy metals	(i) High operating expenses due to low metal removal efficiency (ii) High energy demands (iii) Reduced soil fertility; they are all factors that contribute to secondary pollution

2.	[13, 38]	Treatment with microbes	(i) Passive and active methods of biosorption and bioaccumulation	(i) Reduces energy usage costs while also inhibiting germs (ii) Removing odors (iii) Improving air quality	(i) The process is restricted because of the existence of a nonbiodegradable pollutant (ii) It produces microbial toxicity

3.	[39]	Treatment based on plants	(i) Phytoextraction (ii) Phytostabilization (iii) Phytodegradation	(i) Low cost (ii) Efficient (iii) Simple to maintain and environmentally friendly	(i) Permanence of soil amendments (ii) Metals are leached into groundwater

4.	[6]	Treatment based on nanomaterial	(i) Adsorption (ii) Treatment-based single- stage (iii) Chelation	(i) Surface impact (ii) Quantum effect (iii) Macro quantum effect	(i) The usage of hazardous chemicals, agglomeration (ii) Limited real-life uses and scarcity of comprehensive investigation
